# A decade of advances in the study of buckwheat for organic farming and agroecology (2013-2023)

**DOI:** 10.3389/fpls.2024.1354672

**Published:** 2024-03-06

**Authors:** Yedra Vieites-Álvarez, Manuel J. Reigosa, Adela M. Sánchez-Moreiras

**Affiliations:** ^1^ Universidade de Vigo, Departamento de Bioloxía Vexetal e Ciencias do Solo, Facultade de Bioloxía, Vigo, Spain; ^2^ Instituto de Agroecoloxía e Alimentación (IAA), Universidade de Vigo, Ourense, Spain

**Keywords:** buckwheat, organic farming, agroecology, polyphenols, sustainable weed management

## Abstract

During the last decade, research has shown the environment and human health benefits of growing buckwheat (*Fagopyrum* spp.). This comprehensive review aims to summarize the major advancements made in the study of buckwheat from 2013 to 2023, focusing on its agronomic characteristics, nutritional value, and potential applications in sustainable agriculture. The review examines the diverse applications of buckwheat in organic and agroecological farming systems, and discusses the ability of buckwheat to control weeds through allelopathy, competition, and other sustainable farming methods, such as crop rotation, intercropping and green manure, while improving soil health and biodiversity. The review also explores the nutritional value of buckwheat. It delves into the composition of buckwheat grains, emphasizing their high protein content, and the presence of essential amino acids and valuable micronutrients, which is linked to health benefits such as lowering cholesterol levels, controlling diabetes and acting against different types of cancer, among others. Finally, the review concludes by highlighting the gaps in current knowledge, and proposing future research directions to further optimize buckwheat production in organic or agroecological farming systems. It emphasizes the need for interdisciplinary collaboration, and the integration of traditional knowledge with modern scientific approaches to unlock the full potential of buckwheat as a sustainable crop.

## 1 Introduction

The existence of a crop with allelopathic activity, strong competitive traits against weeds, high protein, vitamin and fiber contents, and antioxidant, anticancer, antihypertension, antibacterial and anti-inflammatory potential sounds unreal, but it is not, is just buckwheat. An emergent functional crop.

### 1.1 Buckwheat

Buckwheat (*Fagopyrum* sp.) is an herbaceous crop belonging to the family Polygonaceaea, whose name comes from the triangular shape of buckwheat seeds. Buckwheat is one of the most unknown alternative gluten-free pseudo-cereal crops originated in the southwest of China, around the mid-6^th^ millennium BC, and spread to Europe from around the 3^rd^ millennium (Hunt et al., 2018). Buckwheat seeds look very similar to a grain, but they are not grains, that’s why buckwheat does not belong to the family Gramineae or Poaceae (Fletcher, 2016). There are 15 species described belonging to the *Fagopyrum* genus, but the two most worldwide cultivated species are the common buckwheat (*Fagopyrum esculentum* Moench.) and the Tartary buckwheat (*Fagopyrum tataricum* Gaertn.) (Džafić and Žuljević, 2022). The morphology of the achene is one of the most important differences between *F. tataricum* and *F. esculentum*. *F. tataricum* achenes are grooved with angles that are rounded in the bottom and sharply acute in the top, whereas *F. esculentum* achenes are not grooved with sharply acute angles (Rangappa et al., 2023). These differences also influence their ability to acclimatize to cold and drought conditions. Morphologically, the stem is usually round and hollow, and frequently changes from green to red. The flowers are usually a cymose cluster that needs to be pollinated to produce seeds. Buckwheat has a long flowering period and serves as a source of nectar for honey. The root system is dense and fibrous, and has a deep main root. Most of the root system is concentrated in the first 25 cm of the soil (Rangappa et al., 2023). Buckwheat has a short life cycle, low nutritional requirements, and abundant biomass production (Pinski et al., 2023), which make this plant an ideal cover crop in organic or agroecological farming systems.

Regarding the nutritional profile of buckwheat, the seeds are rich in protein (much more than cereals), amino acids, and minerals, showing high contents of lysine, tryptophan, arginine, sterols, vitamins, and phenolic compounds (PC). These compounds (PC) are the most important molecules in buckwheat, and are responsible for the strong value of this crop in organic farming and pharmaceutical studies. Among these specialized metabolites is remarkable the flavonoid rutin, which has been reported to show cardioprotective, vasoprotective, antihypertensive, anti-inflammatory, cytoprotective, and anti-diabetic properties (Chrungoo and Chettry, 2021).

Surprisingly, although considered an emergent crop, buckwheat production was about 2,263,764.35 tons in 2013, and the cultivation covered 2,263,608 ha worldwide, while just 7 years later buckwheat production decreased to 187,5067.97 tons and the cultivation covered 1,988,534 ha worldwide in 2021 (https://www.fao.org/faostat/en/#data/QCL), probably related to more than one reason, as discussed by Pirzadah and Rehman (2021), who listed a list of potential reasons including, among others, erratic yield, indefinite growing cycle, tendency for abortion of flowers, sensitivity to freezing conditions, or the presence of allergy-inducing compounds, as well as consumers’ habits and economic limitations. Nonetheless, buckwheat must be still seen as a pseudo-cereal crop with strong potential for functional food sector in the near future.

### 1.2 Weeds in buckwheat agroecosystems

The definition of weed is linked to land uses and human interest. Actually, many definitions of weed have been proposed by the scientific community from various perspectives: agronomic, biological, ecological, etc. The most commonly used definitions have been those provided by the European Weed Research Society in 1986 (“any plant or vegetation, excluding fungi, interfering with the objectives or requirements of people”) and the Weed Science Society of America in 1989 (“a plant growing where it is not desired”) (Scavo and Mauromicale, 2020). Weeds are generally considered annoying, aggressive and competitive. Not only due to the weed-related high yield loses but also by their direct impact on the dietary quality of food, hosting insects, pests and other crop pathogens, decreasing land value and interference with water management (Zimdahl, 2018; Scavo and Mauromicale, 2020).

At the time when these definitions were established, synthetic herbicides began to be used more assiduously with fast and high effectiveness, eliminating weeds, and preventing the loss of crop productivity (Jabran et al., 2015). Unfortunately, due to the massive and indiscriminate use of synthetic herbicides over the last years, many weed species became resistant, avoiding the mode of action of these synthetic chemical compounds and resulting in an important problem, which needs to be urgently resolved. During the last decade, the study of weed resistance has been the focus of several agronomic research studies. The increase of this resistance development has been so fast that while (Heap, 2014) reported, in the international survey of herbicide resistant weeds, 220 herbicide-resistant weeds in 2013, with 404 unique cases (species x site of action) globally, only ten years later, in (Heap, 2023), the number of herbicide-resistant species increased up to 267 with 513 unique cases. Moreover, more than 20% of weeds were found to show multiple resistance, e.g., resistance to different herbicide groups in the same biotype, which complicates even more the control of these species in the agroecosystems.

Regarding buckwheat, the most problematic weed species can vary depending on the region and the specific growing conditions, but there are some of them that are very extended all over Europe, and most of them have showed resistance to some type of synthetic herbicide group. This is the case of *Amaranthus retroflexus* L., which is one of the most harmful weed species to buckwheat and has been reported to show resistance to the synthetic herbicides metamitron (electron transport inhibitor) and fomesafen (protoporphyrinogen oxidase (PPO) enzyme inhibitor) (Adamczewski et al., 2019; Huang et al., 2020); *Chenopodium album* L., which has presented also resistance to metamitron; *Echinochloa crus-galli* (L.) Beauv. that is resistant to acetolactate synthase (ALS) inhibitors (34 cases), acetyl-CoA carboxylase (ACCase) inhibitors (23 cases), photosystem-II inhibitors (11 cases), auxin mimics/cellulose biosynthesis inhibitors (9 cases), very long chain fatty acid inhibitors (6 cases), and microtubule assembly inhibitors (1 case) (Damalas and Koutroubas, 2023); *Bidens pilosa* L., which is resistant to photosystem II (PSII) and ALS inhibitor herbicides such us imazethapyr and atrazine (Takano et al., 2016); *Lolium rigidum* Gaud., which showed resistance to acetyl-CoA carboxylase (ACCase), acetolactate synthase (ASL), photosystem II (PSII), 5-enolpyruvylshikimate-3-phosphate synthase (EPSPS), glutamine synthase, very long-chain fatty acid (VLCFA) synthesis, and protoporphyrinogen oxidase (PPO) inhibiting herbicides (Fernández-Moreno et al., 2017; Loureiro et al., 2017); and *Portulaca oleracea* L., which developed resistance to PSII inhibitors (Okafor et al., 2014).

However, a plant species only becomes a weed if the cultivation conditions and the management of the agroecosystem favor the spread of this species. In fact, in a multi-diverse and balanced agroecosystem, plant species would behave as spontaneous flora, promoting biodiversity and crop development, not behaving as weeds (Zimdahl, 2018). Hu et al. (2023), analyzed in a review the 100 essential questions about the future of agriculture, and identified four Key points to understand the agricultural systems in a holistic way: (i) Resource and Environment, (ii) Agricultural Production, (iii) Nutrition and Health, and (iv) Social and Economic Impacts. These perspectives support that agriculture has a substantial impact on environment, human health, and society, while producing food and other essential goods (such as fiber, biofuels, medicine, etc.). Agriculture relies on the natural resources, which must be sustainably managed to ensure productive and healthy farming systems for long-term viability. Moreover, agricultural production should be effectively and sustainably conducted to maximize yield, while minimizing adverse environmental effects. Agriculture’s main goals are nutrition and health, which include social and economic aspects, as well as the health of humans and the planet (Hu et al., 2023). Therefore, the tomorrow’s agriculture should focus on increasing biodiversity in the agricultural soils (also plant biodiversity), and favoring an autonomous healthy system that can manage the occurrence of a diversity of plants species in the crop fields without damaging crop plants or reducing their yield. Hence, this review proposes some tested and effective agroecological/organic methods to manage spontaneous plant species without the use of herbicides. Alternative weed management strategies will imply a change of paradigm in the current agriculture, implementing a holistic approach that keeps an eye on the whole ecosystem instead of on the plant alone (González-Andujar, 2023).

### 1.3 Agroecological strategies

Agroecology (AE), which emerged almost a century ago, is the application of ecological science to the study and design of sustainable agriculture (Altieri, 1995). AE will allow farmers to increase the power and control of their production while minimizing social and ecological costs from agriculture, such as soil degradation, water contamination, exhaustion of non-renewable resources, susceptibility to climate change, and inequitable social structures (Mier y Terán Giménez Cacho et al., 2018). AE has gained popularity in recent years by appearing as the most comprehensive solution to the many challenges confronting the agri-food systems (Gliessman and Montenegro, 2021). It is regarded as the bedrock for transforming the conventional agri-food systems to more sustainable and resilient agroecosystems, mitigating the environmental impacts on agriculture while ensuring efficiency in the use of natural resources (Gliessman, 2021). AE seeks to integrate (agro) ecology science with agricultural practice and social concerns (Wezel et al., 2020) for the development of sustainable, productive, and resilient agroecosystems.

Different solutions based on agroecological concepts, principles and practices have been developed to preserve productivity and food security in the long-term, while providing ecological benefits and reducing negative external impacts (FAO, 2019; Atta-Krah et al., 2021). Practices carried out in agroecological systems include the selection of crops with allelopathic potential, crops with strong competitive traits against weeds, cover crops, mulching, crop rotation, intercropping, etc.

On the other hand, according to the Agricultural and Rural Development (2023), organic farming aims to produce food using natural preparations and processes. Organic farming has a limited environmental impact by promoting responsible use of energy and natural resources, maintenance of biodiversity, preservation of regional ecological balance, enhancement of soil fertility and maintenance of water quality. Organic farming regulations in the European Union are intended to provide a clear structure for the production of organic goods throughout the EU, and are designed to satisfy consumer demands for reliable organic products, while providing equal opportunities for producers, distributors, and marketers. According to European Commission’s Agriculture and Rural Development, and US-EPA (United States Environmental Protection Agency Integrated pest management (IPM) principles), some of the practices carried out in an organic farming system would include, similarly to agroecological systems:

(I) The screening and selection of naturally resistant varieties to pests and diseases (i.e., allelopathic and/or competitive crops against weeds) would reduce the reliance on synthetic pesticides and promote plant health.
(II) Crop rotation. This sustainable farming method is based on growing different crops on the same land in a planned sequence, which will break pest and disease cycles and will take advantage of allelopathic and competitive traits of crops.
(III) Cover crops and green manures: Cover crops are grown to protect and improve the soil, preventing erosion, suppressing weeds, enhancing soil fertility, and providing habitat for beneficial insects and microorganisms. Green manures, which are cover crops specifically grown to be incorporated into the soil, add organic matter and nutrients.
(IV) Composting: is the process of decomposing organic materials, such as plant residues and animal manure, to create nutrient-rich compost. Organic farmers can use compost to enrich the soil, improve its structure, and enhance nutrient availability for plants.
(V) Soil management: the above-mentioned practices are used to enhance soil health, promoting healthy crop growth and enhancing soil fertility and structure.
(VI) Integrated Pest Management (IPM): organic farmers employ IPM strategies to manage pests and diseases. This approach involves a combination of cultural, physical and mechanical practices, biological control methods, and the use of approved organic pesticides when necessary.

In this context, the goal of this review is synthesizing the advances made in the use of buckwheat for agroecology or organic agriculture systems over the last decade (2013-2023), and providing updated information on the importance and versatility of buckwheat as crop, with the aim of increasing buckwheat production.

## 2 Phenolic compounds: occurrence and function

Phenolic compounds, the most ubiquitous compounds in buckwheat, are present in most of the plant organs of this crop, including fruits, seeds, leaves, stems, roots, etc (De la Rosa et al., 2019). Phenolic compounds are specialized metabolites produced by the phenylpropanoid and shikimic acid pathways, including at least one aromatic ring with one hydroxyl group (Bhatla and Lal, 2018). Currently, more than 8000 phenolic compounds have been identified so far (Vuolo et al., 2019). These specialized metabolites can be divided into different groups according to their skeletal structure (i.e., monomeric, dimeric or polymeric phenolics). For example, flavonoids are polymeric phenolics with a C6-C3-C6 skeleton, while phenolic acids are dimeric phenolics with a C6-C1 skeleton. Other phenolic compounds may present other type of basic skeleton as C6-C3 (hydroxycinnamic acid), C6-C2-C6 (stilbenes), (C6—C3)n (lignins), etc (Lattanzio, 2013).

### 2.1 Flavonoids

Among the phenolic compounds, flavonoids are the most abundant in food and are considered to be the most bioactive phenolics in terms of antioxidant, antihypertensive and anti-inflammatory properties (De la Rosa et al., 2019). The presence of these compounds in buckwheat has been recently reviewed by Matsui and Walker (2020), focusing on their synthesis and their presence in various plant organs of buckwheat. Moreover, a recent study has shown the variation of flavonoids composition on *F. esculentum* (common buckwheat, CB) and *F. tataricum* (Tartary buckwheat, TB), two different species of buckwheat, finding that after sowing both species, rutin tended to decrease in stems, leaves and flowers of CB, while tended to increase in leaves and flowers of TB, although decreased amount of rutin could be found in the stems of this buckwheat species. In fact, the maximum peak of rutin was found in TB flowers 50 days after sowing. Quercetin tended to decrease in the stem of CB and all organs of TB, while increasing in leaves and flowers of CB, with the highest peak of quercetin found in TC flowers 40 days after sowing, similarly to rutin. Finally, quercitrin was only found in CB flowers (Kim et al., 2023).

**Figure 1:**
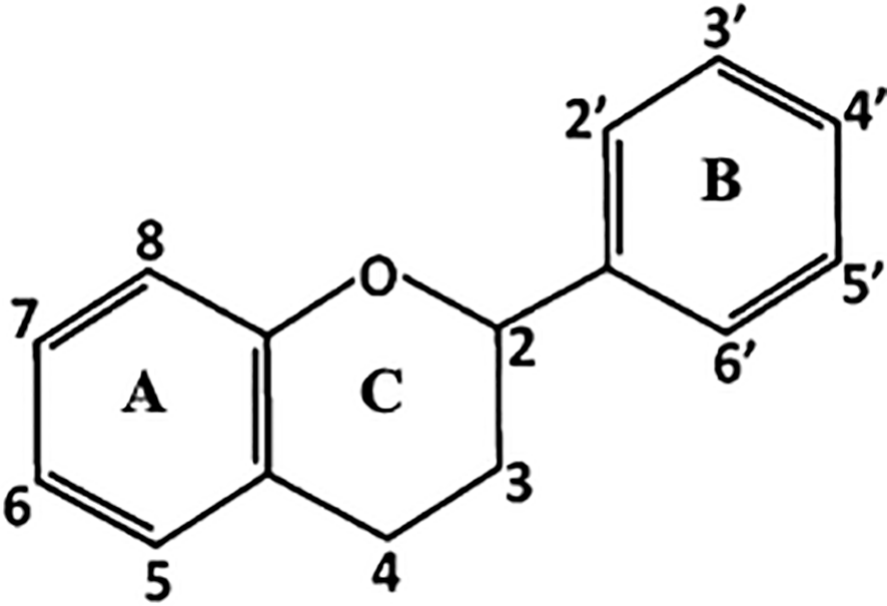
Basic skeleton of flavonoids formed by two phenyl rings **(A, B)** joined through a heterocyclic pyran ring **(C)**.

**Figure 2:**
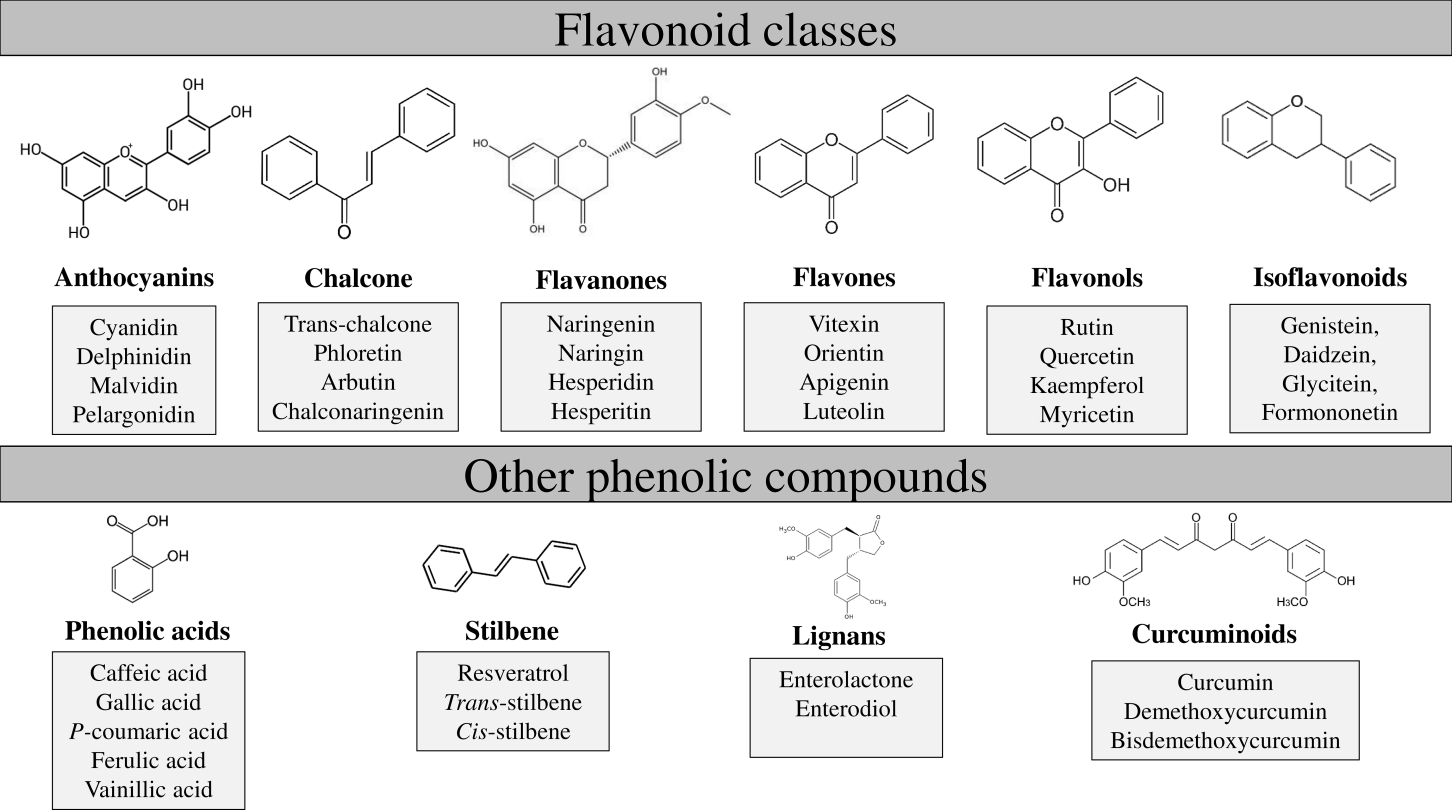
Flavonoids and other phenolic compounds divided in subclasses. Flavonoids include anthocyanins, chalcone, flavanones, flavones, flavanols and isoflavonoids. Other phenolic compounds are phenolic acids, stilbene, lignans and curcuminoids.

The main structure of flavonoids includes two phenyl rings (A and B) joined through a heterocyclic pyran ring (C) (**Figure 1**). Depending on the B-ring position and the degree of unsaturation and oxidation of the C-ring, there will be different types of flavonoids; i.e., isoflavones are flavonoids with the B-ring attached to position 3 of the C-ring, while neoflavonoids have the B-ring attached at position 4 or position 2. This last group can be further subdivided into several subgroups based on the structural features of the C-ring: chalcones, anthocyanins, flavones, flavonols, flavanones, and flavanols (**Figure 2**; Panche et al., 2016).

#### 2.1.1 Isoflavonoids

Isoflavonoids, also known as phytoestrogens, are naturally occurring non-steroidal phenolic plant compounds (Křížová et al., 2019) with the B-ring attached to the third position of C-ring and mostly found in soybean and legumes (Panche et al., 2016). Genistein, daidzein, glycitein, biochanin A, and formononetin are the main types of isoflavonoids (Danciu et al., 2018). These compounds, which have fungistatic, antibacterial, antiviral, and antioxidant properties (Křížová et al., 2019), act as phytoalexins, antimicrobial compounds found to be accumulated in plants after infections, and help to limit pathogen spread (Ahmed and Kovinich, 2021). They also have functions in plant-microbe interactions, including defense mechanisms and rhizobia-legume symbiosis (Rípodas et al., 2013). Ten different isoflavones have been characterized in buckwheat (Li et al., 2019). However, their weed suppression potential has not been well studied (Sohn et al., 2021).

#### 2.1.2 Chalcones

The name chalcones derives from the Greek word “chalcos” and means “bronze”, which is the color of most of the natural chalcones (Zhuang et al., 2017). Chalcones are open chain flavonoids with a 15-carbon structure characterized by the absence of a ‘C-ring’ in the basic flavonoid skeleton structure (Díaz-Tielas et al., 2016). In terms of human health, chalcone has therapeutic activities including anticancer (Mahapatra et al., 2015), antioxidant, anti-inflammatory (Guazelli et al., 2021), antimicrobial (Ušjak et al., 2019), antiulcer (Sashidhara et al., 2015), anthelmintic, antiviral and antiprotozoal activities (Goyal et al., 2021). In plants, chalcones are the precursors of many other phenolic compounds (Rudrapal et al., 2021), and not only play an important role in antioxidant activity, but also the analogues, *cis*-chalcone and *trans*-chalcone have shown strong phytotoxic potential against the development of adventitious species such as *Plantago lanceolata* L., against the early root growth of *A. retroflexus*, and *E. crus-galli*, and against the development and growth of adult *Arabidopsis thaliana* L. Heynh. plants (Díaz-Tielas et al., 2014; Chotsaeng et al., 2019).

**Figure 3:**
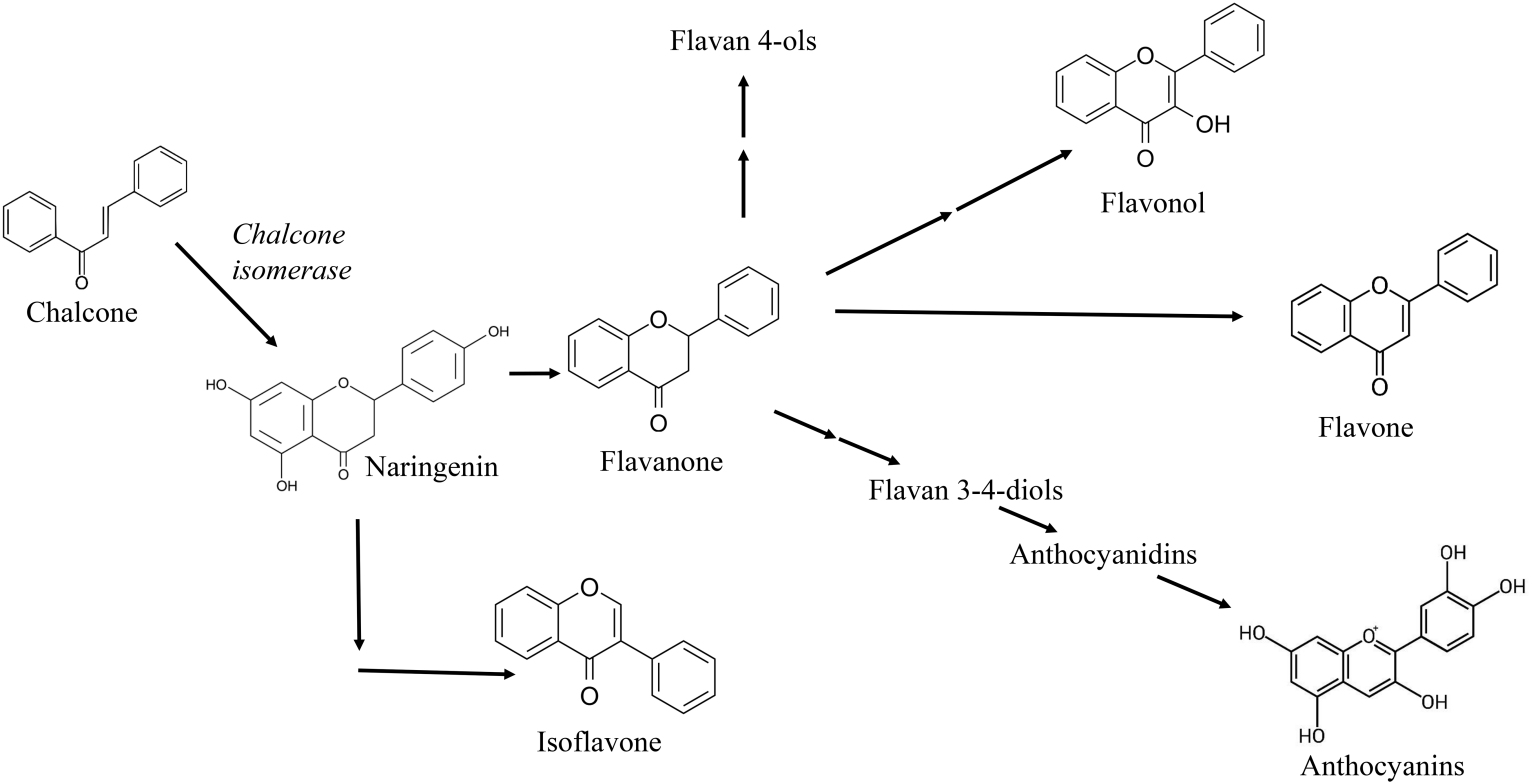
Chalcone as precursor of many flavonoids such as flavone, flavonol, anthocyanidin and isoflavone in plants.

In buckwheat, chalcone related enzymes [i.e., chalcone synthase (CHS) and chalcone isomerase (CHI)] are biogenetic precursors of flavonoids and isoflavonoids (**Figure 3**) (Rammohan et al., 2020), and some of them, as naringenin chalcone, have been related with important antioxidant roles into the buckwheat plant metabolism (Dumitru et al., 2021; Huda et al., 2021). Moreover, CHS gene expression is also increased in buckwheat under stressors like UV light, bacterial or fungal infection, suggesting its antioxidant function (Schenke et al., 2019).

#### 2.1.3 Anthocyanins

Anthocyanins, such as cyanidin, delphinidin, malvidin, pelargonidin, and peonidin, are pigments commonly found in leaves, flowers, and fruits resulting from the hydroxylation, methylation, glycosylation or acylation of the C-ring of the basic skeleton of flavonoids (Alappat and Alappat, 2020). They can be found in outer cell layers of fruits, like berries and red grapes (Panche et al., 2016), and are predominantly found in sprouts and flowers of buckwheat, being considered as the predominant group of polyphenols in pigmentation (Fang et al., 2019). Their functions in plants, as buckwheat, include attracting pollinators (Khan et al., 2022a), UV protection (Costa et al., 2015), antioxidant activity (Tsurunaga et al., 2013), and stress response (Li et al., 2015).

#### 2.1.4 Flavonols

Flavonols are compounds glycosylated in the C-3 position of the C-ring of the basic skeleton (Flamini et al., 2013). The most known flavonols in buckwheat are rutin, quercetin and kaempferol (Barreca et al., 2021). They behave as UV- and photo-protectors, as strongly absorb UV-A and UV-B wavelength (Šikuten et al., 2020). They also participate in the regulation of auxin transport and cellular redox status, being involved in physiological processes as root development and gravitropism (Gayomba et al., 2016), shoot development (Kuhn et al., 2016), stomatal movements (Watkins et al., 2017), and, ultimately, in plant growth. Flavonols also play an important role in antioxidant activities by inhibiting ROS synthesis or by scavenging ROS after oxidative burst (Muhlemann et al., 2018). These metabolites, deeper explained in the section 3 of this review, are the most predominant in buckwheat, and are the main responsible of its pharmaceutical potential.

#### 2.1.5 Flavanones

Flavanones, also present in buckwheat, are generally present in all citrus fruits such as oranges, lemons and grapes, and are responsible of the bitter taste of juice and peel of citrus fruit (Zhao et al., 2020). Hesperitin, naringenin and eriodyctiol are examples of this class of flavonoids (**Figure 2**). In particular, naringenin is a very special molecule in buckwheat, not only because plays a key role as precursor of other flavonoids, such as quercetin, rutin, kaempferol etc (Marín et al., 2018), but also because these flavanones confer potential human health benefits such us antioxidant, anticancer, anti-inflammatory, blood lipid-lowering and cholesterol-lowering (Panche et al., 2016; Stabrauskiene et al., 2022). In fact, it has been recently characterized as a promising treatment strategy against COVID‐19 due to its antiviral and anti‐inflammatory effects (Tutunchi et al., 2020). Regarding the importance in plants, flavanones play essential roles in insect-plant interactions, pigmentation, heavy metal tolerance, disease resistance, and UV-protection (Khan and Dangles, 2014).

#### 2.1.6 Flavanols

Flavanols are a subclass of flavonoids with a hydroxyl group on either C-3 or C-4. There are four main types: flavan-3-ols, flavan-4-ols, isoflavan-4-ols, and flavan-3,4-ols. The most common flavanols in buckwheat are catechin and epicatechin, followed by procyanidin (Janovská et al., 2021). Regarding human health, flavanols have been shown to improve vascular function through the enhance of endothelial function, resulting in the improvement of blood flow and the reduction of inflammation in the blood vessels (Al-Dashti et al., 2018), and in cognitive functions (Pa and Gazzaley, 2014). In plants, flavanols play an important role in defense against pathogens and herbivores through antimicrobial activity properties (Medic et al., 2021), flower pigmentation, UV protection by absorbing harmful UV radiation, and as ROS scavengers avoiding DNA damage and oxidative stress (Csepregi and Hideg, 2018). Flavanol content in leaves is inversely proportional to anthocyanins content, and flowers with high flavanol contents are mainly cream-white (Nakayama, 2020; Han et al., 2023).

#### 2.1.7 Flavones

Flavones are characterized by their chemical structure, which includes a backbone of 2-phenylchromen-4-one. The name “flavone” is derived from the Latin word “*flavus*”, referring to the yellow pigmentation often associated to these compounds (Deryabin et al., 2019). Flavones are widely present in leaves, flowers and fruits as glucosides. Apigenin, luteolin, vitexin and orientin are the most common flavones in buckwheat (Janovská et al., 2021). Like other flavonoids, flavones serve to a variety of purposes that help plants to adapt to different biological environments, such as (i) defense against UV radiation and oxidative stress; (ii) interspecies interactions (pathogen resistance, symbiosis, defense against herbivory, and allelopathy); and (iii) plant development (co-pigmentation with anthocyanins, and lignification); among others (Jiang et al., 2016).

### 2.2 Other phenolic compounds

Phenolic acids, stilbenes, lignans, tannins, and curcuminoids are phenolic compounds structurally different from flavonoids. They are characterized by the absence of the flavonoid structure, and typically contain a single phenol ring or other unique structures. While most of the phenolic compounds in this group have smaller and simpler chemical structures than flavonoids, there are some phenolic compounds with complex structure and high molecular weight, as well. Phenolic acids have one carboxylic group and one or more OH groups instead of the single phenyl group (Tsimogiannis and Oreopoulou, 2019). Phenolic acids can be further classified into two main groups: hydroxycinnamic acids (derived from cinnamic acid) and hydroxybenzoic acids (derived from benzoic acid). Examples of hydroxycinnamic acids include ferulic, caffeic, and *p*-coumaric acids, while examples of hydroxybenzoic acids include gallic and protocatechuic acids, both very ubiquitous in buckwheat (Koval et al., 2020), contributing to the color, flavor, and nutritional properties of this pseudo-cereal. Moreover, phenolic acids are known for their antioxidant activity. They can scavenge free radicals and other reactive oxygen species, helping to protect cells from oxidative damage. Phenolic acids have been also associated with various health benefits, as anti-inflammatory, antimicrobial, and anticancer properties (García-Pérez et al., 2013; Embuscado, 2015; Gonçalves et al., 2017).

## 3 Most abundant phenolic compounds in buckwheat

Nowadays, buckwheat is mainly cultivated for its versatile and highly nutritious seeds, which are used for gluten free bread, pasta, tea, flour, etc. However, the cultivation and study of seedlings and adult plants (sprouts, shoots and roots) have gained importance in the recent years due to their high content of phenolic compounds (PCs), which are very beneficial for plant, animal and human health (Karki et al., 2013; Dziadek et al., 2018). The most abundant PCs found in the two major cultivated species of buckwheat, *F. esculentum* and *F. tataricum*, are flavonoids and phenolic acids (Mansur et al., 2022).

### 3.1 Rutin

**Figure 4:**
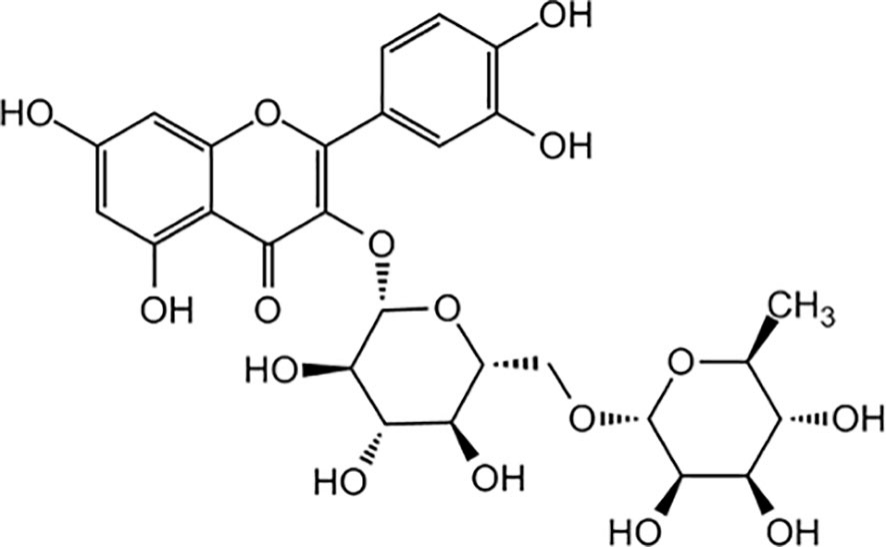
Chemical structure of rutin (quercetin + disaccharide rutinose).

The most abundant PC in buckwheat is the flavonol rutin, found in both species, but specially in *F. tataricum* (Mikulajová et al., 2016; Vieites-Álvarez et al., 2023a). Rutin is a glycoside of the flavonoid quercetin, as is a combination of this flavonol with the hydroxyl group at position C-3 substituted with glucose and rhamnose sugar groups (disaccharide rutinose) (**Figure 4**). Polekhina (2013) revealed that rutin is mainly found in the leaves and flowers of buckwheat plants (between 63.2 to 76.5% of the total amount of rutin in a plant) followed by the stems (from 7 to 15.7%), and finally the roots (from 1.7 to 5%), depending on the variety. Borovaya and Klykov (2020) found a similar pattern of accumulation, with large amounts of rutin in flowers of *F. esculentum* (47 to 63 mg g^-1^), significantly less in stems (6 to 14 mg g^-1^), and the minimal amount in roots (3 to 8 mg g^-1^). Moreover, Kalinová et al. (2019) also found great amounts of rutin in the embryo axis with the cotyledons.

Rutin has shown great benefits for human health such as antioxidant, antihypertensive and anti-inflammatory compound (Nishimura et al., 2016). Into the plant, rutin is involved in the reduction of environmental stress, UV-light protection, antioxidant activity and disease resistance (Suzuki et al., 2015). Vollmannová et al. (2013) studied the amount of rutin, and its antioxidant activity, in the pseudo-cereals buckwheat, quinoa and amaranth, and showed that the large amount of rutin found in buckwheat is probably correlated to the strong antioxidant activity of this crop, which is higher than quinoa or amaranth. In the same way, Lee et al. (2016) found that rutin and orientin contents in buckwheat are directly correlated to its antioxidant properties, which are closely linked to the attenuation of the impact of environmental stress on plant metabolism. Song et al. (2022) tested two *F. tataricum* varieties (named TR and CG) at different altitudes, and found that the synthesis of rutin in TR was upregulated as response to cold stress. Moreover, rutin is also considered a strong phytotoxic molecule. Ladhari et al. (2020) showed that high levels of rutin inhibited the growth of *Lactuca sativa* (L.), *Raphanus sativus* (L.), *Peganum harmala* (L.) and *Silybum marianum* (L.). The exposure of monocotyledonous and dicotyledonous plants such as alfalfa, cress, lettuce, rapeseed, barnyard grass, foxtail fescue, Italian ryegrass, and timothy to aqueous extracts of *Cassia alata* (L.), a plant species with high rutin content, resulted in the growth inhibition of shoots and roots for all the species (Das et al., 2019). Additionally, rutin, together with chlorogenic acid, were responsible for the inhibition of the growth of *Allium cepa* L (Fonseca et al., 2017).

### 3.2 Quercetin

**Figure 5:**
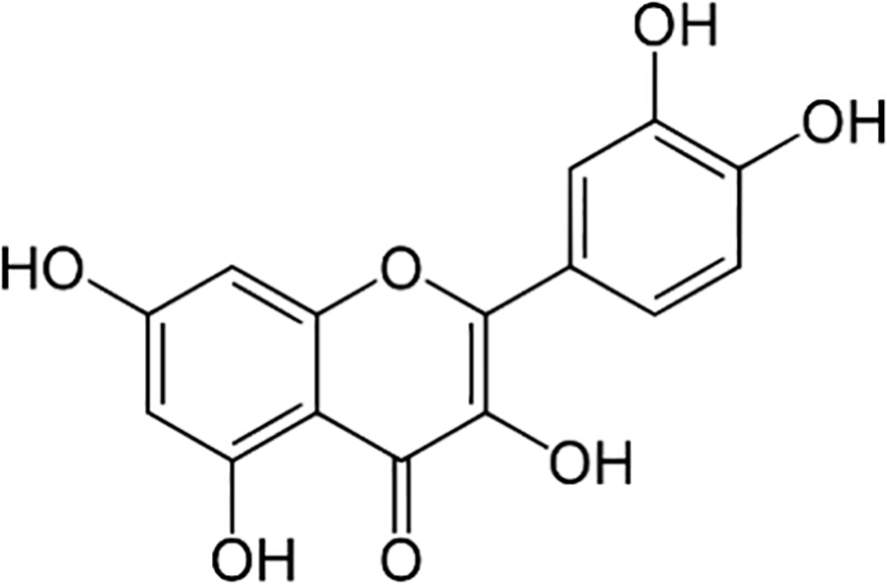
Quercetin chemical structure.

Quercetin, the precursor of rutin, is other important PC in buckwheat. Quercetin is a type of flavonol with a hydroxyl group on the third carbon of the C-ring (**Figure 5**) that is mainly found in seeds and cotyledons of buckwheat, especially in Tartary buckwheat. Suzuki et al. (2005) found a negative correlation between rutin and quercetin contents, as quercetin is an intermediate compound in the rutin biosynthesis in plants. In this sense, Ren and Sun (2014) observed that quercetin content started to decrease five days after sprouting, when rutin content started to increase. Quercetin participates in numerous physiological processes in plants, including photosynthesis, pollen development, antioxidant machinery, and seed germination (Singh et al., 2021). Kurepa et al. (2016) discovered that *A. thaliana* treated with quercetin reduced the amount of ROS produced by the ROS-producing herbicide paraquat, suggesting that quercetin would be acting as an antioxidant. Ng et al. (2015) and Tohge et al. (2016) also attributed to quercetin the ability to promote healthy plant growth and development by controlling auxin transport, limiting cell proliferation, and promoting the cell elongation phase. Due to its potent antioxidant properties, quercetin effectively protects plants from a variety of biotic and abiotic stress factors (Singh et al., 2021). Quercetin has also shown potential to interfere with the development of other species. This flavonoid induced the inhibition of germination of alfalfa seeds and the reduction in the weight and length of alfalfa seedlings (Ghimire et al., 2019). Moreover, quercetin is very effective inhibiting root development. Fernández-Aparicio et al. (2021) found that root growth of *Phelipanche ramose* (L.) Pomel was inhibited by *F. esculentum* root exudates with high content of quercetin. Coelho et al. (2017) observed that quercetin extracts had the same effects in primary roots of leguminous plants. Additionally, Chen et al. (2019) and Zhao et al. (2023) discovered that quercetin has potential to affect photosynthesis, respiration, cell membrane, and enzymatic system of *Microcystis aeruginosa* Kutzing’s, causing oxidative damage in a concentration-dependent way.

### 3.3 Phenolic acids

Phenolic acids, including chlorogenic, caffeic, vanillic, ferulic, *p*-coumaric, gallic, dihydroxybenzoic, syringic, protocatechuic, salicylic, phthalic and *m*-toluic acids, among others, are also very important compounds in buckwheat tissues (Vollmannová et al., 2021). The function of phenolic acids in plants is very diverse, being involved in various aspects of plant growth, development, and defense. Phenolic acids serve as signaling molecules; especially salicylic acid (Kobyletska et al., 2022), which plays a role in plant-microbe interactions (Mandal et al., 2010) and contribute to plant resistance responses to stress (Sytar et al., 2014). In general, phenolic acids show antioxidant properties acting as protective agents against oxidative stress (Wiczkowski et al., 2016). Additionally, these specialized compounds are involved in regulating enzymatic activity, influencing growth and development processes, attracting pollinators and seed dispersers, and providing UV protection (Cheynier et al., 2013; Marchiosi et al., 2020). Therefore, these versatile compounds are essential for plant survival and adaptation to their environment. Most of them has shown potential to manage surrounding weeds. According to Hoang-Anh et al. (2021), the mode of action of phenolic acids could involve the induction of oxidative stress in target plants, the modification of cell division and permeability, the alteration of photosynthesis, respiration, and transpiration, and the modulation of gene expression, protein biosynthesis, phytohormone activities, and enzyme functions. For example, Fu et al. (2019) observed allelopathic effects of phenolic acids on seedling growth and photosynthesis of *Rhododendron delavayi* Franch, and found that ferulic, chlorogenic and protocatechuic acids were highly effective inhibiting stomatal activity (opening, conductance, density and length and width), and reducing the photosynthetic rate. As well, they demonstrated that chlorogenic acid significantly inhibited conductance and transpiration rate in *R. delavayi* seedlings. Leaf extracts of *Juglans regia* containing, among other compounds, protocatechuic and caffeic acids, were able to inhibit *Amaranthus retroflexus* L. and *C. album* L. germination and seedling growth (Đorđević et al., 2022). Moreover, leaf litter leachates of different alfalfa cultivars with high content of *p*-hydroxybenzoic, caffeic, *p*-coumaric, chlorogenic, ferulic and cinnamic acids, inhibited seedling growth of *Festuca arundinacea* (Schreb.) Darbysh, and *Sorghum sudanense* (Piper) Stapf (Wang et al., 2017).

## 4 Using buckwheat in agroecology and organic farming for weed management

The fast establishment of buckwheat in the environment, its high canopy growth, and the presence of phenolic compounds with antioxidant but also allelopathic properties, makes of buckwheat a very interesting crop to be exploited on sustainable agricultural such us organic farming and agroecology (Sturm and Gerhards, 2016). These types of farming are focused on the production of food without the use of synthetic fertilizers, pesticides, other artificial chemicals or genetically modified organisms (GMOs) (International Federation of Organic Agriculture Movements IFOAM, 2020). Organic farming is linked to several benefits to environment and human health (Durán-Lara et al., 2020), being an environmentally sustainable solution to prioritize the protection of soil health, water quality, and biodiversity (Siddique et al., 2014). Beyond the same purpose, agroecology entails the application of the science of ecology to the study, design, and management of sustainable food systems, the integration of the diverse knowledge systems generated by food system professionals, and the involvement of the social movements that are promoting the transition to fair, just, and responsible food systems (Anderson et al., 2019).

In this context, the buckwheat’s inherent properties make it an ideal crop for organic farming and agroecology, as its growth and specialized metabolites favor soil health and quality while sustainably managing weeds. On the one hand, due to its deep and fibrous root system, buckwheat roots help to improve soil structure, increasing porosity and allowing better infiltration of water and air (Hudek et al., 2021). On the other hand, the fast shoots development helps to avoid wind and water erosion by providing a surface cover and improving water infiltration. Moreover, this property is also related to the addition of organic matter to the soil through decomposition of its biomass (Mpanga, 2022). Its short flowering period promotes increased biodiversity as buckwheat flowers are able to attract a variety of pollinators, such as bees and butterflies, which contribute to the pollination of other nearby crops and can provide shelter and food for beneficial insects, such as pest predators, helps to maintain a natural balance in the agricultural ecosystem (Płażek et al., 2023). Finally, buckwheat can help in weed control in organic farming. Due to its rapid growth, dense cover and high presence of specialized compounds in its tissues, which can behave as allelochemical compounds through root exudates, leaf leachates or litter decomposition, buckwheat can compete with weeds, reducing their growth and spread (Falquet et al., 2015).

### 4.1 Allelopathic potential of buckwheat for weed management

Allelopathy, defined by Rice (1984) in 1984, refers to both inhibitory and stimulatory reciprocal biochemical interactions between plants and microorganisms including any direct or indirect effect (by microorganisms) of one plant on another through the production of chemical compounds. Allelopathy plays an important role in agroecosystems through a wide variety of interactions between plants and other organisms (Aslam et al., 2017).

The screening of allelopathic buckwheat cultivars has been of great importance during the past decade. Allelopathic crops naturally exude specialized metabolites (phenolic acids and flavonoids) that can be useful for sustainably manage surrounding weeds, helping to reduce the use of synthetic herbicides by farmers (Aslam et al., 2017).

The allelopathic effects of buckwheat are attributed to the compounds present in the plant, including flavonoids, phenolic acids, fatty acids, and other allelochemicals (Li et al., 2019). The release of these compounds to the medium can affect the growth and development of surrounding weeds (Vieites-Álvarez et al., 2023a, Vieites-Álvarez et al., 2023b).

Buckwheat plants start to release allelopathic compounds during germination and early development (Kalinová et al., 2005). Zou et al. (2014) also found that allelochemicals released by buckwheat in an early phase of development, induced the growth inhibition of couch grass (*Elymus repens* L.), showing the weed-suppressive ability of buckwheat trough root exudates. As well, Gfeller et al. (2018) showed that common buckwheat could alter its root exudation after recognizing the presence of *A. retroflexus*, suppressing this weed’ growth. Fernández-Aparicio et al. (2021) indicated that buckwheat root exudates, rich in the flavonoid quercetin and the stilbene *p-*coumaric, were able to induce the inhibition in *Phelipanche ramosa* (L.) Pomel radicles. More recently, Vieites-Álvarez et al. (2023b) tested the allelopathic potential of four buckwheat varieties (Kora, Eva, PI481671 and Gema) from two different species (*F. esculentum* and *F. tataricum*), and found that buckwheat plants growing in co-culture with the weeds *L. rigidum* or *P. oleracea* released to the medium much higher amounts of flavonoids (i.e., rutin, quercetin, orientin, vitexin, etc.) and phenolic acids (i.e., ferulic acid, protocatechuic acid, syringic acid, etc.) during the first days of development (7-17 days), than buckwheat plants growing alone, which favored the inhibition of weeds’ growth. These allelochemicals present in different tissues are often extracted to test their inhibitory potential on weeds.

Mioduszewska et al. (2013) evaluated water extracts from common buckwheat (from aboveground and roots tissues), and found that aboveground extracts showed stronger inhibition in the germination of lettuce seeds than root extracts. Uddin et al. (2013) observed that 7.5 mg mL^-1^ Tartary buckwheat extract reduced the growth of *Galium spurium* L. up to 33.7%. Recently, Szwed et al. (2020) showed that a very low percentage (1%) of the aqueous extract of 14-day common buckwheat plants was able to inhibit root development of wild oat (*Avena fatua* L.), yellow foxtail (*Setaria glauca* L. P. Beauv.), barnyardgrass (*Echinochloa crus-galli* (L.) Beauv.), common windgrass (*Apera spica-venti* (L.) P. Beauv.), catchweed bedstraw (*Galium aparine* L.), scentless mayweed (*Matricaria inodora* L.) and gallant soldier (*Galinsoga parviflora* Cav.), and the shoot length of wild oat. As well, more recently (Cechin et al., 2023), three concentrations of extracts from seeds or shoots of buckwheat (25, 50 and 100%) resulted in the reduction of germination and abnormal seedling development of the weeds *B. pilosa* and *Euphorbia heterophylla* L., with *B. pilosa* being more sensitive than *E. heterophylla* to both extracts of buckwheat.

This phytotoxic activity of buckwheat is probably due to the PCs present in buckwheat tissues, which can be accumulated in shoots or roots or relocated to be released to the medium (Vieites-Álvarez et al., 2023c). In other research, gallic acid and (+)-catechin, extracted from buckwheat shoots, were found to be the responsible for the inhibition of roots and shoots of lettuce seedlings (Iqbal et al., 2017). A recent study demonstrated that methanolic extracts of gallic, caffeic and ferulic acids in concentrations of 153.96, 69.13, and 39.80 ppm, respectively, were enough for inhibiting the growth of *Chenopodium murale* L. and *A. viridis* L (Alsharekh et al., 2022). Šćepanović et al. (2022) evaluated the phytotoxic potential of several PCs (chlorogenic, caffeic, ferulic, gallic, protocatechuic, *p*-hydroxybenzoic, syringic, vanillic, and *p*-coumaric acids) on *Ambrosia artemisiifolia* L. and found that treatment with ferulic acid, vanillic acid, *p*-coumaric acid, *p*-hydroxybenzoic acid, or a mixture of all phenolic acids, highly inhibited early growth parameters, suggesting the strong phytotoxic potential of these compounds and the higher toxicity of phenolic acids mixtures. The research of Gfeller et al. (2018) proposed that these compounds would be the responsible for the suppression caused by common buckwheat to pigweed, goosefoot and barnyard grass growth by 53, 42 and 77%, respectively, as they observed significant growth inhibition in an experiment without physical root interactions.

In this context, the screening and selection of varieties with strong allelopathic potential, both through root exudates of living plants or through metabolites present in plant tissues, could provide an alternative solution to the use of synthetic herbicides, as it has been shown that buckwheat has the ability to sustainably manage competing surrounding weeds. Furthermore, the proven effectiveness of these specialized metabolites makes buckwheat a very suitable crop for other agroecological cultivation methods such as mulching, cover cropping, intercropping and green manure.

### 4.2 Competition role in weed management

Although, nowadays, there are numerous studies on the potential of allelopathy or allelochemicals to manage weeds, it is very difficult, under natural conditions, to differentiate between allelopathy and competition, as both occur simultaneously in plant communities and co-exist in the agroecosystems influencing weed germination and development. Laboratory tests usually do not take into consideration the soil environment and the multiple abiotic conditions that crops and weeds cope with during their development in the agroecosystems. Moreover, specialized metabolites undergo continuous changes under natural conditions depending on the physical, chemical and biological conditions of the soil, which will determine their phytotoxic level. Therefore, the interaction of weeds and crops in communities is a complex combination of competition for resources and the effect of plant specialized metabolites (Bączek and Halarewicz, 2022).

Regarding resource competition, weeds can compete with crops for water, nitrogen, light, nutrients and space (Sardana et al., 2017). The establishment of plants in the environment depends heavily on phenotypic traits like shoot and root length, leaf number, and plant height (Kunstler et al., 2016). Crop accessions with a higher initial growth rate and a quicker canopy development rate can establish more quickly and will be less impacted by weed competition (Dass et al., 2017). Sturm et al. (2018) evaluated the relative proportion of allelopathic effects with respect to weed inhibition. They used *F. esculentum* as a cover crop with and without activated charcoal in the soil (used as adsorbent of allelopathic substances), and concluded that although allelopathy contributed significantly to the overall suppression of weeds, competition had a bigger impact. These authors concluded that to increase the effectiveness of weed control, an allelopathic cover crop should show competitive prerequisites, as rapid germination, fast development, dense canopy, and high soil coverage.

Common buckwheat is one of the fastest growing cover crops used in Western Europe. Seedlings emerge in less than one week, and cover 40–80% of the soil within 4 weeks after sowing, producing around 200 g m^−2^ of shoot and root dry matter. For this reason, buckwheat is considered one of the best cover crops (Brust et al., 2014; Kunz et al., 2016).

During the past decades, some authors suggested that light competition could be the most important factor for weed suppression in buckwheat fields, as the rapid development of buckwheat canopy prevents weed establishment due to lack of light (Tominaga and Uezu, 1995; Bicksler and Masiunas, 2009). However, there has been no experimental evidence for this hypothesis so far. In 2013, Gebhard et al. (2013), demonstrated that the amount of crop biomass produced (i.e., the amount of shading), was closely correlated to the ability of legumes to compete with weeds. Brust et al. (2014) conducted a field experiment with cover crops such as white mustard, oilseed radish, forage radish, phacelia, Tartary buckwheat, red oat, and grain amaranth, and a no cover crop control to identify the weed suppression ability of cover crops. They found that Tartary buckwheat was the cover crop with the highest shoot growth eight weeks after sowing, resulting in more than 95% weed suppression. Zou et al. (2014) concluded that the high leaf area index (LAI) of buckwheat could be one of the weed-suppressive factors decreasing *E. repens* growth and lowering its weight dry mass. As well, Smith et al. (2020) obtained a 98% weed suppression in a buckwheat monoculture field due to its cover soil ability.

Moreover, competition not only takes place aboveground. Below-ground competition for resources constitutes also an important aspect of crop-weed interaction. This type of competition is naturally occurring for space, soil nutrients and water, as plants mainly take up nutrients and water from the proximal root zone (Asaduzzaman et al., 2014). Falquet et al. (2014) performed an experiment in pot trials in order to detect competition in soil between two varieties of buckwheat and redroot pigweed. They found that the roots of redroot pigweed were up to 87% smaller after growing in co-culture with the buckwheat varieties than after growing alone in the same space (no influence of space limitation). Recently, Vieites-Álvarez et al. (2023a) showed that some buckwheat varieties could modify their growth resulting in longer roots after co-cultivation with monocot or dicot weeds. The ability to modify root length could be an advantage in water uptake competition. In this sense, Martínez-Goñi et al. (2023) tested the behavior of wheat (*Triticum aestivum* L.), spelt (*Triticum spelta* L.), and common buckwheat (*F. esculentum*) under well-watered conditions, mild drought an extreme drought, and found that buckwheat was the only crop able to cope with extreme drought conditions by increasing its water use efficiency and maintaining its photosynthetic parameters. This property of buckwheat is, and in the future will be even more, an advantage in increasingly common and widespread periods of drought due to climate change (Yuan et al., 2023).

### 4.3 Allelopathy and competition interaction in weed management strategies (cover crops, mulching, intercropping, crop rotation and green manure)

The combination of allelopathic and competitive traits in buckwheat as a cover crop may enhance its weed suppressive capacity (Florence et al., 2019), making this pseudo-cereal suitable for other weed management methods such as intercropping, crop rotation or green manuring. These techniques are often indicated as distinct and separate methods, but they can be considered as sister strategies, as all are based in the same buckwheat properties (Scavo and Mauromicale, 2021). In fact, cover crops, as buckwheat, can be applied as living mulches, when intercropped with industrial crops; as dead mulches, when plant residues are left on the surface of the soil; or as green manures, when the residues are incorporated into the soil (Scavo and Mauromicale, 2021). Unfortunately, these techniques have not been further studied in buckwheat during the last decade, although all are promising strategies for the establishment of agroecological approaches in agricultural production, which should be taken into consideration in the near future.

#### 4.3.1 Mulching

Mulching is the practice of applying a layer of material on top of the soil to provide various benefits to plants and the overall soil. Mulch can be made from a variety of organic or inorganic materials, such as grass clippings, straw, bark chips, stones, plant residues, or plastic. The benefits of mulching include weed suppression, moisture retention, soil temperature control, erosion prevention, nutrient cycling and improved soil structure (Khan et al., 2022b). Generally, living mulching is a more effective method for controlling weeds than non-living mulching (i.e., cover crop residues), as the first one can suppress weeds throughout the growing season while the second option is more effective in preventing weed seed germination and smothering very young weeds (Westbrook et al., 2022). Mulches can manage weeds by mixing competition (light, water, and nutrients) and allelopathy. This is a very efficient method for light interception, which compromises germination and growth of many weeds. Moreover, reduction in light intensity is also correlated with modifications in soil temperature, which can also delay the germination of weeds (Batlla and Benech-Arnold, 2014). Mulches can also modify water availability by transpiration, which could have greater impacts on shallow-rooted weeds than deeper-rooted crops (Westbrook et al., 2022). Regarding nutrient competition, buckwheat has a high carbon-to-nitrogen (C:N) ratio, which means that it can potentially tie up nitrogen in the soil and temporarily reduce its availability for other plants meanwhile provides a large amount of C to soil (Vecchia et al., 2020).

Besides, allelopathy plays also an important role in mulching. Buckwheat allelochemicals are present in different plant tissues, as leaves, flowers, shoots and roots, but they are not necessarily released to the medium, accumulating in the different organs of the plant. After the plant cycle, the plant residues can be left in the soil or can be dried to straw. The rapid leaf development of buckwheat allows obtaining more dry matter in less time, making it ideal for this agroecological technique. Root residues from buckwheat were able to inhibit biomass of barnyard grass (*E. crus-galli*) and cleavers (*Galium aparine* L.) in a dose-response way when left on the soil (Szwed et al., 2019), with different mixtures inducing different weed management potential. The mixture of 0.5 t ha^-1^ buckwheat with 1.0 t ha^-1^ marsh pepper residues was the most effective in suppressing weeds while improving grain yields (Afroz et al., 2018). Mulches of *F. tataricum* tested in a field with *C. album* L., *Matricaria chamomilla* L. and *Stellaria media* (L.) Vill induced a decrease in germination rate and dry matter of those weeds respect to untreated fields (Sturm et al., 2016). Straw of buckwheat inhibited weed development in broccoli fields too, probably due to the combination of the physical properties of the mulches (color, structure, etc.) and their allelopathic effects (Kosterna, 2014a). Latify et al. (2017) proposed that an adequate selection of sunflower competitive varieties in combination with buckwheat living mulch could be the best option to manage weeds on sunflowers fields, as they observed a reduction in the density of weeds (up to 96%) when compared with a control yield (without buckwheat mulches and non-competitive sunflower variety). Moreover, buckwheat helps protecting radish and cucumber crops by suppressing diseases, when added to the soil at least three weeks before planting (Abbasi et al., 2018). Pfeiffer et al. (2015), after a research involving *F. esculentum* mulch and snap beans (*Phaseolus vulgaris* var. Tavera) with bell peppers (*Capsicum annuum* var. Revolution) and fall broccoli (*Brassica oleracea* var. Imperial) as cultivated plants, stated that there is an inverse relation between living mulch biomass and weed biomass, confirming the weed suppression ability of living mulches, although yield productivity could be sometimes reduced due to resource competition among plant species, living mulches and weeds.

Finally, buckwheat mulching not only can be useful for weed control. In conventional cropping systems, buckwheat mulching successfully prevented leaching of herbicides (such as diclosulam and diuron) to the soil by creating a chemical barrier which involves the prevention of soil contamination (Munhoz-García et al., 2023). Moreover, it was observed that after covering the field with buckwheat residues, the daily temperature fluctuations were reduced, and the moisture increased at depths between 0 - 40 cm, improving soil broccoli production compared to a field without mulching (Kosterna, 2014b).

The combination of the two characteristics previously mentioned (presence of phenolic compounds in buckwheat tissues + relevant leaf biomass) makes of buckwheat an ideal crop for mulching.

#### 4.3.2 Intercropping

Intercropping is a strategy defined by the simultaneous growth of two or more crops in the same field over an extended period of time, without necessarily sowing or harvesting them at the same time (Bedoussac et al., 2015; Li et al., 2020). Intercropping is an eco-functional practice that has been extensively used to increase crop productivity (Qin et al., 2013) and land use efficiency (Kidane et al., 2017), while reducing the quantity of greenhouse gases emitted to the atmosphere when compared with monoculture’ fields (Naudin et al., 2014). When allelopathic crops are grown in intercropping systems, allelochemicals are released into the environment through root exudation, volatilization from above or below-ground plant parts, leaching from rainfall, or decomposition of plant debris (Scavo et al., 2018). By increasing the diversity of the soil’s microbial population and facilitating the transport of allelochemicals’ into the soil, intercropping could improve the allelopathic weed–cover crop interactions and, as a result, the phytotoxic effects (Brooker et al., 2015).

Biszczak et al. (2020) found that soybean intercropped with buckwheat (i): 1 row of soybean + 1 row of buckwheat, or (ii): 1 row of soybean + 2 rows of buckwheat, reached a significant reduction of total weed number and weeds’ dry weight when compared to mono-cultured soybean fields. Although the highest soybean yields were obtained in intercropping with lentils, it is necessary to choose crops that have a balance between yield improvement and weed control. Cheriere et al. (2020) found that soybean intercropped with sunflower (2018) and buckwheat (2019) provided the best weed control, while ensuring high soybean yields. Salehi et al. (2018) intercropped fenugreek and buckwheat in a 2:1 ratio, improving fenugreek productivity. Very recently, Lakshmi et al. (2023) found that intercropping buckwheat with maize, baby corn, sweet corn and sunflower would work well, and even the microbial activity of the soil may vary depending of the crop mixture.

However, intercropping is not always a good idea. Žalac et al. (2022) checked land and water productivity (WP) in an old and dense orchard compared to a younger one, both with intercropping buckwheat or barley and walnut trees, and found a reduction in walnut yields and WP due to competition between crops in old orchards, although buckwheat productivity reached the highest yield and WP in the young orchard. Smith et al. (2020) compared the efficacy of intercropping six cover crops (with a total of 14 species mixtures) and monoculture these crops to control weed abundance (weed biomass) and to reach weed suppression during three seasons: summer (spring planting-summer termination), fall (summer planting-fall termination), and spring (fall planting-subsequent spring termination). They found that, irrespective of the season, mixtures were never more weed suppressive than the most weed-suppressive cover crop grown as a monoculture. For example, buckwheat monoculture reached a weed suppression of 97-98% in summer season, while the mixture of buckwheat with other crops was less effective ranging from 66 to 96%. Similar patterns were also found for other cover crops, suggesting that farmers could reach better results of weed suppression in monoculture systems.

#### 4.3.3 Crop rotation

Crop rotation is a traditional agricultural practice that offers numerous benefits for weed and pest control, reduced auto-allelopathy and nutrient leaching, improved soil organic matter and soil fertility, and increased crop yields (Scavo and Mauromicale, 2021). Root exudates and decomposing crop residues from allelopathic buckwheat crops enriched the soil with allelochemicals that favored weed control in the next crop cycle (Jabran et al., 2015). Buckwheat grows well with other early-maturing crops like potatoes, spring greens, or winter canola. Crop rotation of buckwheat–buckwheat–potatoes resulted in 30% less N leaching and 16% more tuber yield (Jiang et al., 2022), maybe due to buckwheat roots containing acyl sucrose, which causes hormonal imbalances in wireworms, whose larvae cause extensive damage to potatoes (Alkhnajari, 2019).

#### 4.3.4 Green manure

Green manure is a technique that takes advantage of cover crops by harvesting and mixing them with soil to provide valuable organic matter and weed control compounds. Buckwheat has shown to be very useful as green manure due to its allelopathic properties and the ability to incorporate nutrients to the soil, especially phosphorous and nitrogen (Boglaienko et al., 2014). Masilionyte et al. (2017) tested the weed-suppressive potential of buckwheat mixed with other cover crop as white mustard (*S. alba*), both cultivated for green manure in a 6-year field experiment. The number of weeds and the weed biomass were significantly lower in buckwheat-mustard mixed fields than after the cultivation of narrow-leafed lupine (*Lupinus angustifolius* L.) in a mixture with oil radish (*Raphanus sativus* L.), thanks to its biomass production and release of allelochemicals into the soil. Reddy et al. (2021) obtained increased plant growth, dry-matter accumulation, tillers, grain yield, and economic improvement in rice (*Oryza sativa* L.) under green-manuring with buckwheat.

## 5 Cultivation practices for improving buckwheat potential in weed management

Buckwheat is a crop that can be cultivated under different growing conditions, although different buckwheat species developed different in a diverse range of environments (Rangappa et al., 2023). Making a selection of the buckwheat cultivars according to the geographical area, weather conditions, need for tillage, or seeding distance could enhance the buckwheat ability to growing better and managing more successfully adventitious plants.

### 5.1 Weather conditions

The temperature, quantity of rainfall and sunshine hours will affect the total biomass production and grain yield and quality of buckwheat (Jung et al., 2015). Even when buckwheat can increase water use efficiency under extreme drought conditions (Martínez-Goñi et al., 2023), this pseudo-cereal is sensitive to high temperatures and hot dry winds, especially under low humidity conditions (Dražić et al., 2016). However, different species of buckwheat are genetically regulated to better withstand different climatic situations. *F. tataricum* is more resistant to cold due to a DNA methylation (Song et al., 2020), and more resistant to drought than *F. esculentum*, while this species thrives better on sandy and well-drained soils (Dražić et al., 2016). So, Tartary buckwheat is regarded as a potential crop for cultivation at higher altitudes because of its adaptability to various climatic variables and water-stress regimes, cold temperatures, and nutrient-deficient acid soil (Rangappa et al., 2023). Temperature and altitude are also one of the most determinant environmental factors in the production of flavonoids as rutin (Sobhani et al., 2014; Dražić et al., 2016), which will determine the allelopathic properties of buckwheat. Regarding rainfall, buckwheat is highly susceptible to dryness, particularly in early growth stages, during rooting, flowering, and yielding period. However, moisture excess during the later stages of growth has also strong detrimental effects on buckwheat development (Nikolic et al., 2019).

### 5.2 Sowing time

The timing of this activity is quite related to temperature. Sowing time is one non-monetary input that significantly influences crop productivity (Chaudhary et al., 2019), and buckwheat is not an exception. Sowing time influences seed germination, flowering time, length of vegetation, rutin content, and herb and grain yield of buckwheat (Babu et al., 2018). The range for seed germination varies from 5 to 42°C, but the optimum temperature is around 24-26°C (Dražić et al., 2016). Buckwheat seeds must be sown at different times, depending on the geographic area, to avoid flower blasting, which usually occurs at temperatures higher than 32°C (Płażek et al., 2019). Once flowering has started, the plants should be at least 10 weeks in non-frost weather conditions. Rangappa et al. (2023) obtained the highest mean yield of buckwheat when the sowing date was between October and December in the Himalaya region, while in central New York, Björkman and Shail (2013) determined that the optimal timing for sowing buckwheat was late June to early August, and in Korea, Jung et al. (2015) proposed mid to end of August as the best time for sowing buckwheat.

### 5.3 Tillage requirement

To decide on using tillage or a non-till system will mainly depend on the characteristics of the planting soil. Buckwheat, as a low input crop that can be cultivated under reduced tillage system (Chrungoo and Chettry, 2021). Actually, buckwheat requires low-fertility soil with a moderate nitrogen content to start growing (Gairhe et al., 2015). Low tillage can boost buckwheat crop germination and establishment by creating a seedbed that facilitates optimal seed-to-soil contact. Additionally, soil’s incorporation of organic matter and crop residue can be improved also by tillage, which can enhance the soil’s fertility and structure. Tillage can also control the growth of adventitious plant species avoiding the emergence of weeds and burying weed seeds (Myers, 2018). Nevertheless, buckwheat can be drilled without tillage, which is a viable choice especially for mid-summer planting. This strategy can reduce soil erosion and help preserving soil moisture. Additionally, weed management can also be favored by tillage, because the residue from the previous crop acts as a mulch to prevent the growth of weeds. The particular characteristics of the field, such as the type of the soil, the amount of moisture, and the pressure from weeds, should be taken into account when determining whether or not tillage buckwheat fields (Myers, 2018).

### 5.4 Seed rate, sowing depth and spacing between rows

The optimal seed rate of buckwheat can vary from 90 to 160 grains per m^2^ as maximum (Nikolic et al., 2019; Wan et al., 2023) because higher seedling rates can result in puny plants with short stems (Tolaini et al., 2016), which would impede the proper plant development, thus minimizing the ability of buckwheat to control weeds in an agroecological system. A proper plant population is the key for reaching higher crop yields, as plants could use all available resources without inter-cultivar competition (Babu et al., 2018). Depth of sowing is also crucial for determining the ideal plant population. Gerhards and Schappert (2020) proposed 2–3 cm as a good seeding depth for all cover crop species if a mixture is sown. This allows small and large-seeded cover crops to emerge. Xiang et al. (2014) tested three different sowing depth in *F. tataricum* (2, 4 and 6 cm), and concluded that buckwheat seeds sown at 4 cm had an emergence rate, seedling number, seedling rate, stem diameter, dry matter, area per plant, and leaf chlorophyll content significantly higher than when seeds were sown at 2 or 6 cm. Regarding row spacing, there are some controversial mainly depending on the species. Kolarić et al. (2021) determined that 25 cm instead of 50 cm spacing between rows resulted in higher average in grain yield for *F. esculentum*. However, Salmankhan et al. (2021) found that closer spacing of 30 cm between rows × 10 cm between plants resulted in taller plants, while wider row spacing of 45 between rows × 15 cm between plants resulted in higher number of leaves.

Finally, depending on the intended purpose, the time of harvest may also vary. In a previous study (Kara, 2014) where buckwheat was harvested at different stages of the crop (1^st^ harvest stage (HS): the beginning of flowering; 2^th^ HS: 20 days after 1^st^ harvest (around the full blooming period); 3^rd^ HS: 32 days after 1st harvest (about the end of flowering), 4^th^ HS: 45 days after 1^st^ harvest (about 25% of grains had turned brown) and 5^th^ HS: 55 days after 1^st^ harvest (about 50% of grains had turned brown), Kara (2014) concluded that harvesting time may vary depending on the final interest. For higher plant matter and crop yields (i.e., for mulching, seed production, etc.), buckwheat should be harvested 55 days after sowing, however, for strong mineral nutrient content in plant organs, the plants should be harvested 20 days after sowing (for pharmaceuticals, food sector, etc.).

## 6 Buckwheat nutritional profile and health

Buckwheat, in terms of nutrition, has been established as one of the most complete and nutritious foods. Buckwheat grains are a rich source of protein with a balanced amino acid composition, gluten free flour, dietary fiber, vitamins, resistant starch, phytosterols, fagopyrins, fagopyritols, and phenolic compounds (Sofi et al., 2023). Some slight differences are observed in the nutritional composition of different buckwheat species (**Table 1**).

**Table 1:** Differences in the nutritional composition of common buckwheat (*Fagopyrum esculentum*) and Tartary buckwheat (*Fagopyrum tataricum*).

Parameters	Protein	CH	Lipid	Dietary fiber	Ash	Other
Common buckwheat ^a^	12.30	54.50	3.80	7.00	2.00	18.40
Tartary buckwheat ^a^	13.15	57.40	3.84	10.6	2.70	10.53

CH, carbohydrates; Other refers to other compounds such soluble carbohydrates, organic acid, nucleotides, and other unknown compounds. Data are given in %.

a Adapted and modified from Collar and Angioloni (2014)

In addition to the high vitamin and mineral content, buckwheat differs from other foods by its high content of phenolic compounds, which were already found to have a positive effect on human health. The phenolics that can be found in buckwheat are well described in the section 1 of this review. The main responsible molecules of this biological activity in buckwheat are rutin and quercetin, both found in higher amounts in *F. tataricum* than in *F. esculentum.* These flavonols are correlated to antioxidant, anticancer, antihypertension, antibacterial and anti-inflammatory activities.

Rutin has demonstrated to be a potent antioxidant in several processes in human health. One of the most important functions of rutin is the ability to reduce oxidative stress and inflammatory responses by scavenging ROS or preventing their formation (Mehta et al., 2021), which is directly correlated with its antioxidant properties. Rutin, together with ascorbic acid, showed antioxidant, anti-inflammatory and antiapoptotic activities against UV-induced skin damage, suggesting that both compounds are potentially cytoprotective (Gęgotek et al., 2019). This flavonoid is also a promising neuroprotective compound that may help to fight with neurodegenerative diseases by reducing proinflammatory cytokines and improving antioxidant enzyme activities, among other processes (Enogieru et al., 2018). This antioxidant activity is closely linked to their anti-cancer property. Rutin participates in the inhibition of proliferation, attenuation of superoxide production, and decrease adhesion and migration of human cancerous cells (Sghaier et al., 2016), showing ability to inhibit the proliferation of breast (Yang et al., 2017), colon (Alonso-Castro et al., 2013), renal (Caparica et al., 2020), lung (Imani et al., 2021), and prostate (Ding et al., 2017) cancer, as well as other tumors by the regulation of several signal pathways. Moreover, together with quercetin, rutin has the ability to prevent liver inflammatory injury (Lee et al., 2013), and antimicrobial synergistic effects with antibiotic activity against drug-resistant bacterial (Alnour et al., 2022).

Quercetin has also shown antidiabetic (Rezabakhsh et al., 2019), anti-inflammatory (Rafiq et al., 2015), antioxidant (Leyva-Soto et al., 2021), antimicrobial (Nguyen and Bhattacharya, 2022), anti-Alzheimer’s (Joshi et al., 2022), and cardiovascular (Dabeek and Marra, 2019) activities in humans.

Phenolic acids, including hydroxybenzoic and hydroxycinnamic acids, are the specialized aromatic metabolites that confer typical organoleptic characteristics to foods. They are related to several human health benefits including antioxidant, anti-inflammatory (Shahidi and Zhong, 2015; Tresserra-Rimbau et al., 2018), immunoregulatory, anti-microbial, anti-thrombotic, cardioprotective, anti-cancer (Anantharaju et al., 2016; Mondal et al., 2021) and antidiabetic activities (Kumar and Goel, 2019). Phenolic acids can act delaying microbial growth and inhibiting lipid oxidation, prolonging food shelf-life (Galanakis, 2018). In deep, each phenolic acid can behave in a different way. For example, hydroxybenzoic acid could act as a mediator in colorectal cancer prevention (Sankaranarayanan et al., 2020) and as strong antioxidant potential (Das et al., 2022), while gallic acid shows hyperlipidemic, antihyperglycemic, cardioprotective and anti-cancer potential (Zanwar et al., 2014; Sourani et al., 2020), protocatechuic acid demonstrated antioxidant, antimicrobial, anti-inflammatory, antiapoptotic and antiproliferative activity (Semaming et al., 2014), vanillic acid has activity as hepatoprotective, cardioprotective, anti-apoptotic, anti-proliferative, and against DNA-induced damage (Almeida et al., 2016), caffeic acid has antioxidant and antibacterial properties (Spagnol et al., 2019; Possamai-Rossatto et al., 2021), and chlorogenic acid was reported to improve cognitive function in elders (Kato et al., 2018).

The high content of phenolic compounds in buckwheat makes it a highly nutritious and disease-protective crop.

## 7 Future areas of research

Future areas of research in buckwheat encompass various aspects of its cultivation, breeding, and utilization:

(i) *Breeding for improved traits*: research can focus on developing buckwheat varieties with enhanced traits such as disease resistance, tolerance to abiotic stress factors (e.g., drought, heat), improved nutritional content, and optimized agronomic characteristics, as increased pest control potential.
(ii) *Molecular information*: functional genomics could be applied to identify genes involved in biosynthesis of several identified phenolics, in order to achieve the isolation of these compounds.
(iii) *Climate resilience*: with changing climatic conditions, studying the adaptability of buckwheat to different environments and its response to drought, heat, and other climate-related stress factors can be valuable for future cultivation.
(iv) *Utilization and value-added products*: exploring the potential of buckwheat for applications such as functional foods, nutraceuticals, and industrial uses, can open up new avenues for its commercialization and utilization.
(v) *Enhancing collaboration* between farmers, weed scientists and plant breeders to advance in the breeding and exploitation of this crop, especially in agroecology systems for autonomous weed management.

## Author contributions

YV-Á: Conceptualization, Investigation, Writing – original draft, Writing – review and editing. MR: Funding acquisition, Resources, Supervision, Writing – review and editing. AS-M: Project administration, Resources, Supervision, Writing – review and editing.
